# A multicenter phase II trial of ramucirumab plus irinotecan for early recurrence of gastric cancer during or after adjuvant chemotherapy with docetaxel plus S-1 therapy: the RAMIEL trial (OGSG1901)

**DOI:** 10.1007/s10120-026-01799-8

**Published:** 2026-09-16

**Authors:** Toshifumi Yamaguchi, Ryohei Kawabata, Yusuke Akamaru, Hidekazu Hirano, Keijiro Sugimura, Yasuhiro Miyazaki, Yoshihiro Kakeji, Yosuke Kito, Takashi Nomura, Takeshi Kawakami, Masahiro Tanemura, Atsushi Takeno, Hiroyuki Okuda, Tomohiro Matsushima, Toshio Shimokawa, Yukinori Kurokawa, Shunji Endo, Narikazu Boku, Taroh Satoh

**Affiliations:** 1 Cancer Chemotherapy Center, Osaka Medical and Pharmaceutical University Hospital, 2-7, Daigaku-Machi, Takatsuki, Osaka 569-8686 Japan; 2 Department of Surgery, Sakai City Medical Center, 1-1-1 Ebaraji-Cho, Sakai-Shi, Osaka 272-1199 Japan; 3 Department of Gastroenterological Surgery, Osaka Rosai Hospital, 1179-3 Nakasonecho, Kita-Ku, Sakai-Shi, Osaka 591-8025 Japan; 4 Department of Gastrointestinal Medical Oncology, National Cancer Center Hospital, 5-1-1 Tsukiji, Chuo-Ku, Tokyo 104-0045 Japan; 5 Department of Surgery, Kansai Rosai Hospital, 3-1-69 Inabaso, Amagasaki, Hyogo 660-8511 Japan; 6 Department of Surgery, Osaka General Medical Center, 3-1-56 Bandaihigashi, Sumiyoshi-Ku, Osaka City, Osaka 558-8558 Japan; 7 Division of Gastrointestinal Surgery, Department of Surgery, Kobe University Graduate School of Medicine, 7-5-1 Kusunoki-Cho, Chuo-Ku, Kobe, 650-0017 Japan; 8 Department of Medical Oncology, Ishikawa Prefectural Central Hospital, 2-1 Kuratsuki Higashi, Kanazawa City, Ishikawa Prefecture 920-8530 Japan; 9 Department of Surgery, Yamagata Prefectural Central Hospital, 1800 Aoyanagi, Yamagata-Shi, Yamagata 990-2292 Japan; 10 Division of Gastrointestinal Oncology, Shizuoka Cancer Center, 1007 Shimonagakubo, Nagaizumi-Cho, Sunto-Gun, Shizuoka, 411-8777 Japan; 11 Department of Surgery, Rinku General Medical Center, 2-23 Rinku Orai-Kita, Izumisano-City, Osaka 598-8577 Japan; 12 Department of Surgery, NHO Osaka National Hospital, 2-1-14 Hoenzaka, Chuo-Ku, Osaka, Osaka 540-0006 Japan; 13 Department of Medical Oncology, Keiyukai Sapporo Hospital, 1-1, Minami 9-Chome, Hondori, Shiroishi-Ku, Sapporo-Shi, Hokkaido 003-0026 Japan; 14 Department of Gastroenterology, Saitama Cancer Center, 818 Komuro, Ina-Machi, Kitaadati-Gun, Saitama, 362-0806 Japan; 15 Department of Biostatistics, Faculty of Medicine, Wakayama Medical University, 811-1 Kimiidera, Wakayama-Shi, 641-8509 Japan; 16 Department of Gastroenterological Surgery, Graduate School of Medicine, The University of Osaka, 2-2 Yamadaoka, Suita, 565-0871 Japan; 17 Department of Digestive Surgery, Kawasaki Medical School, 577 Matsushima, Kurashiki City, Okayama 701-0192 Japan; 18 Department of Oncology and General Medicine, IMSUT Hospital, Institute of Medical Science, University of Tokyo, 4-6-1, Shirokanedai, Minato-Ku, Tokyo 108-8639 Japan; 19 Center for Cancer Genomics and Precision Medicine, The University of Osaka Hospital, 2-2 Yamadaoka, Suita, 565-0871 Japan

**Keywords:** Gastric cancer, Early recurrence, Docetaxel, S-1, Irinotecan, Ramucirumab

## Abstract

**Background:**

There is currently no established chemotherapy regimen for early recurrence of pathological stage III gastric cancer (GC) following adjuvant chemotherapy with docetaxel plus S-1 (DS) after D2 gastrectomy. We aimed to evaluate the efficacy and safety of ramucirumab plus irinotecan in patients with GC who experienced early recurrence during or within 6 months after DS adjuvant chemotherapy.

**Methods:**

This prospective, open-label, multicenter phase II trial enrolled eligible patients treated at 25 centers of the Osaka Gastrointestinal Cancer Chemotherapy Study Group in Japan. Patients received ramucirumab (8 mg/kg) and irinotecan (150 mg/m^2^) every 2 weeks. The primary endpoint was overall survival (OS), and secondary endpoints included progression-free survival (PFS), objective response rate (ORR), and safety. The sample size was set at 40 based on a threshold median OS of 7 months and an expected median OS of 11 months, with a one-sided alpha error of 0.05 and a power of 0.80.

**Results:**

Between November 2019 and July 2023, 43 patients were enrolled, and 39 were included in the analysis after excluding three ineligible patients and one who did not initiate protocol treatment. The median OS was 15.9 months (95% CI: 8.8–42.0; *p* = 0.003). The median PFS was 5.5 months (95% CI: 3.9–8.1), and the ORR was 38.5%. Grade  ≥ 3 adverse events, including neutropenia (25.6%) and hypertension (25.6%), were observed in more than 20% of patients.

**Conclusion:**

Ramucirumab plus irinotecan demonstrated promising efficacy and manageable toxicity in patients with early recurrence of GC following adjuvant DS therapy.

**Trial registration:**

This study was prospectively registered in the Japan Registry of Clinical Trials (jRCTs05119071, October 6, 2019, https://jrct.niph.go.jp/latest-detail/jRCTs051190071).

**Supplementary Information:**

The online version contains supplementary material available at https://doi.org/10.1007/s10120-026-01799-8.

## Introduction

Gastric cancer (GC) remains one of the leading causes of cancer-related mortality worldwide, particularly in East Asia [1]. For patients with pathological stage III GC, adjuvant chemotherapy following curative resection is the standard approach to reduce recurrence and improve survival. The ACTS-GC and CLASSIC trials established S-1 and capecitabine plus oxaliplatin as adjuvant therapies, whereas the recent START-2 trial demonstrated the superiority of docetaxel plus S-1 (DS) over S-1 monotherapy in relapse-free survival, thereby positioning DS therapy as a standard treatment for stage III disease [2–6].

Despite curative resection followed by adjuvant chemotherapy, some patients experience early recurrence. Notably, in the START-2 trial, approximately 10% of patients in the DS therapy group experienced relapse during or within 6 months after completing DS therapy [7]. Despite the poor prognosis of patients with early recurrence [8–11], evidence to guide their treatment remains limited. Furthermore, based on retrospective reports indicating poor efficacy, the Japanese GC treatment guidelines advise against reusing adjuvant chemotherapeutic agents for early recurrence [12, 13]. As no standard therapeutic approach is available for patients with early recurrence after adjuvant DS chemotherapy, where fluoropyrimidines and taxanes are not considered active, there is an urgent need for effective alternative chemotherapy regimens.

In advanced GC, ramucirumab (RAM), a VEGFR-2 monoclonal antibody, and irinotecan (IRI) are effective second-line chemotherapeutic agents [14, 15]. The RAINBOW trial demonstrated a significant survival benefit of RAM plus paclitaxel over paclitaxel alone, while the RAMIRIS trial reported improved survival with RAM plus FOLFIRI in patients with metastatic GC [16, 17], suggesting a potential benefit of RAM–IRI combination therapy. Supporting this notion, a Japanese phase Ib trial demonstrated acceptable safety of RAM–IRI in heavily pretreated patients with GC. Similarly, the phase II HGCSG 1603 trial reported promising efficacy and safety of RAM–IRI as a second-line therapy for advanced GC [18, 19]. However, no studies have investigated the potential benefit of this regimen in patients with early recurrence of GC after adjuvant chemotherapy.

The aim of this phase II trial was to evaluate the safety and efficacy of RAM–IRI, with a focus on overall survival (OS), in patients with GC who experienced recurrence during or within 6 months after adjuvant DS chemotherapy for pathological stage III disease.

## Material and methods

### Trial design

**Fig. 1:**
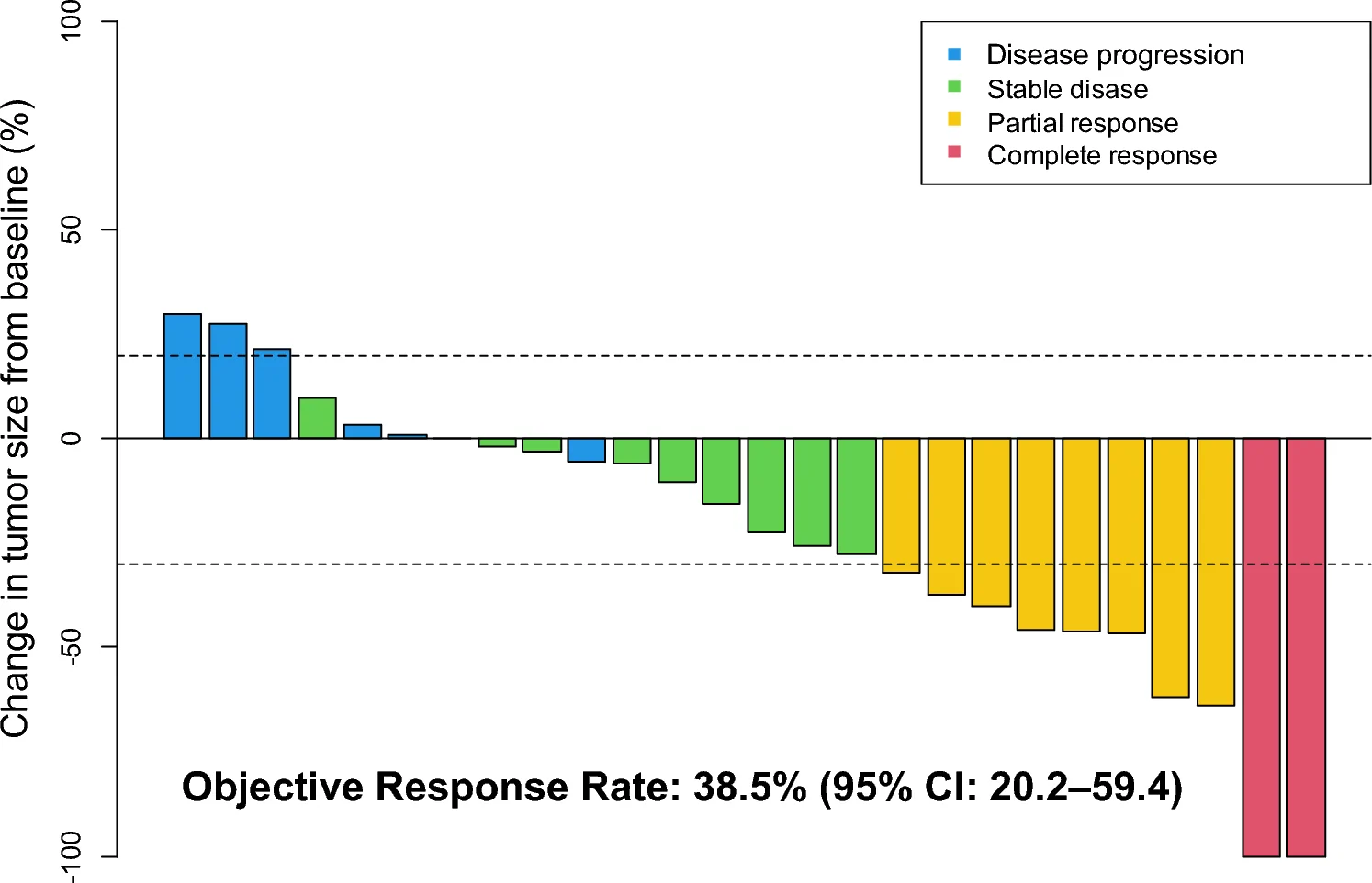
Waterfall plot of the percentage change in tumor size over the course of treatment with ramucirumab plus irinotecan. CI, confidence interval

This prospective, open-label, multicenter phase II trial (OGSG1901) was conducted at 25 centers of the Osaka Gastrointestinal Cancer Chemotherapy Study Group (OGSG) in Japan (Fig. 1). Early postoperative recurrence was defined as: (1) recurrence during adjuvant DS chemotherapy or (2) recurrence within 6 months after completion of adjuvant DS chemotherapy. The study was conducted in accordance with the Declaration of Helsinki and the Japanese Clinical Trials Act. The protocol was approved by the Certified Review Board of the Osaka International Cancer Institute (CRB5180012) and registered in the Japan Registry of Clinical Trials on October 11, 2019 (jRCTs051190071). Written informed consent was obtained from all participants prior to enrollment.

### Patients

The eligibility criteria were as follows: (1) histologically confirmed adenocarcinoma of the stomach or gastroesophageal junction; (2) receipt of adjuvant DS chemotherapy for pathologically confirmed stage III (including ypIII) GC after curative gastrectomy; (3) early recurrence during adjuvant DS chemotherapy (after at least one administration of docetaxel) or within 6 months after its completion; (4) age ≥ 20 years; (5) Eastern Cooperative Oncology Group (ECOG) performance status of 0 or 1; (6) presence of at least one evaluable lesion on computed tomography (CT) or magnetic resonance imaging, either measurable or non-measurable according to Response Evaluation Criteria in Solid Tumors (RECIST) version 1.1; (7) adequate organ function, including a neutrophil count ≥ 1500/mm^3^, hemoglobin ≥ 9.0 g/dL, platelet count ≥ 100,000 cells/mm^3^, aspartate aminotransferase and alanine aminotransferase ≤ 100 IU/L (≤ 200 IU/L in patients with liver metastases), total bilirubin ≤ 1.5 mg/dL (except for grade 1 Gilbert’s syndrome), serum creatinine ≤ 1.5 mg/dL or creatinine clearance ≥ 40 mL/min, urinary protein ≤ 1+ by dipstick or < 2 g/24 h, prothrombin time–international normalized ratio ≤ 1.5, and activated partial thromboplastin time ≤ 5 s above the upper limit of normal; (8) provision of written informed consent; (9) human epidermal growth factor receptor type 2 (HER2)-negative or unknown HER2 status; and (10) no prior treatment with IRI or RAM.

The exclusion criteria encompassed the following: (1) synchronous or metachronous malignancies within 5 years (except carcinoma in situ); (2) symptomatic interstitial pneumonia or pulmonary fibrosis; (3) active infection requiring treatment; (4) serious concomitant illness; (5) pregnancy, breastfeeding, or refusal to use adequate contraception; (6) psychiatric disorders interfering with study participation; (7) ongoing systemic corticosteroid therapy; (8) severe comorbidities; (9) anticoagulant therapy for thromboembolism; (10) uncontrolled hypertension (> 150/90 mmHg despite standard medication); (11) uncontrolled diarrhea; (12) symptomatic central nervous system metastases; (13) blood transfusion within 2 weeks prior to registration; (14) moderate or severe pleural effusion or ascites; (15) treatment with atazanavir sulfate, which is contraindicated with IRI; or (16) any condition deemed inappropriate for study participation by the investigator.

### Treatment

Patients received RAM (8 mg/kg) and IRI (150 mg/m^2^) intravenously on day 1 every 2 weeks. Although uridine diphosphate glucuronosyltransferase 1A1 (UGT1A1) testing was not mandatory, the IRI dose was reduced to dose level  − 1 (120 mg/m^2^) in patients known to have homozygous or double heterozygous *UGT1A1* polymorphisms. If grade 4 neutropenia, grade 2 thrombocytopenia, grade ≥ 3 febrile neutropenia, or grade 3 non-hematological toxicity causally related to IRI occurred, the IRI dose was reduced to the next lower dose. RAM was discontinued in cases of grade ≥ 3 infusion reactions or life-threatening RAM-related adverse events. If urine protein was ≥ 2 +  on dipstick,  ≥  2 g/day proteinuria, or grade 3 RAM-related adverse events (excluding hypertension) were observed, the RAM dose was reduced. RAM administration was interrupted in cases of ≥ 3 g/24 h proteinuria or grade 4 hypertension. Treatment was continued until disease progression, unacceptable toxicity, or withdrawal of consent. Palliative radiotherapy was permitted during the study; however, no other anticancer or investigational drugs were allowed.

### Endpoints

The primary endpoint was OS, defined as the time from registration to death from any cause. Secondary endpoints included progression-free survival (PFS), time to treatment failure (TTF), objective response rate (ORR), disease control rate (DCR), and safety. Tumor assessments, using computed tomography (CT) scans of the chest, abdomen, and pelvis, were performed within 4 weeks before registration and every 8 weeks thereafter. Adverse events were graded according to the Common Terminology Criteria for Adverse Events (CTCAE v5.0). The neutrophil-to-lymphocyte ratio (NLR), prognostic nutritional index (PNI), and modified Glasgow Prognostic Score (mGPS) were evaluated as markers of systemic inflammation and nutritional status. The PNI was calculated as 10 × serum albumin (g/dL) + 0.005 × total lymphocyte count (/mm^3^), and reflected the patients’ nutritional and immune status. The mGPS was based on serum C-reactive protein (CRP) and albumin levels: 0 for a CRP level of  ≤ 1.0 mg/dL, 1 for a CRP level of  > 1.0 mg/dL with an albumin level of  ≥ 3.5 g/dL, and 2 for a CRP level of  > 1.0 mg/dL with an albumin level of  < 3.5 g/dL. Both indices were used as markers of systemic inflammation and nutritional status. Subgroup analyses of OS were performed according to PNI and mGPS categories. Relative dose intensity (RDI) was calculated as the ratio of the cumulative delivered dose to the planned cumulative dose.

### Statistical analysis

Based on the WJOG4007 trial and a multicenter retrospective study by Shitara et al. [13, 15], the null and alternative hypotheses for median OS were set at 7.0 and 11 months, respectively. A total of 39 patients were required to achieve a power of 0.80 with a one‐sided α of 0.05, assuming a 2-year enrollment period and 1.5 years of follow-up after final enrollment. Accordingly, the target sample size was set at 40. Survival outcomes were estimated using the Kaplan–Meier method, with 95% confidence intervals (CIs) calculated using the Brookmeyer–Crowley method. The population for the analysis of treatment effectiveness and safety was defined, in accordance with the study protocol, as all eligible patients who received at least one dose of the study treatment. Statistical analyses were performed at the OGSG Data Center using R software (version 4.4.0; R Foundation for Statistical Computing, Vienna, Austria).

## Results

### Patient characteristics

Between November 2019 and July 2023, 43 patients were enrolled across 25 institutions. The primary analysis included 39 patients, after excluding three ineligible patients (two who had not received adjuvant DS chemotherapy following radical surgery for pathological stage III disease, and one with HER2 positivity), as well as one patient who did not initiate protocol treatment. Online Resource 1 shows the CONSORT diagram of the trial. Patient characteristics are summarized in Table 1.

**Table 1:** Patient characteristics

	Ramucirumab plus irinotecan (n = 39)
Age, years, median (range)	68 (40–81)
Sex, n (%)	
Male	32 (82.1%)
Female	7 (17.9%)
ECOG performance status, n (%)	
0	24 (61.5%)
1	15 (28.5%)
Site of primary tumor, n (%)	
Gastric GEJ	32 (82.1%) 7 (17.9%)
Patological stage, n (%)	
IIIA	12 (30.8%)
IIIB	13 (33.3%)
IIIC	14 (35.9%)
Pathological subtype, n (%)	
Intestinal Diffuse	15 (38.5%) 24 (61.5%)
HER2 status, n (%)	
Negative Unknown	30 (76.9%) 9 (23.1%)
UGT1A1, n (%)	
Wild	15 (38.5%)
Single hetero	13 (33.3%)
Homo/Double hetero	1 (2.6%)
Unknown	10 (25.6%)
Recurrence site, n (%)	
1 ≥ 2	26 (66.7%) 13 (33.3%)
Time of recurrence, n (%)	
During adjuvant DS After adjuvant DS	12 (30.8%) 27 (69.2%)
Comorbidities, n (%)	
Present Absent	12 (30.8%) 27 (69.2%)

DS, Docetaxel plus S-1; ECOG PS, Eastern Cooperative Oncology Group Performance Status; GEJ, gastroesophageal junction; HER2, human epidermal growth factor receptor 2; UGT1A1, uridine diphosphate glucuronosyltransferase 1A1

The median age was 68 (range, 40–81) years, and 32 patients (82.1%) were male; the ECOG performance status was 0 in 61.5% of participants. The primary tumor site was the esophagogastric junction in seven patients (17.9%) and the stomach in 32 patients (82.1%). HER2 status was negative in 30 patients (76.9%), and unassessed in nine patients (23.1%). The *UGT1A1* genotype was wild-type in 15 patients (38.5%), single heterozygous in 13 (33.3%), double heterozygous or homozygous (DH/Homo) in one (2.6%), and not assessed in 10 (25.6%). The pathological stage at radical resection was distributed as follows: IIIA in 12 patients (30.8%), IIIB in 13 (33.3%), and IIIC in 14 (35.9%). Recurrence involved a single organ in 26 patients (66.7%) and two or more organs in 13 patients (33.3%) (Online Resource 2). Histologically, 15 patients (38.5%) had the intestinal type and 24 (61.5%) had the diffuse type. Recurrence was diagnosed during adjuvant chemotherapy in 12 patients (30.8%) and within 6 months after completion in 27 patients (69.2%). Regarding inflammatory, nutritional, and immunological status, the mean NLR was 2.28 ± 1.16, the PNI was 45.40 ± 5.60, and the mGPS was 0 in 28 patients (71.8%), 1 in eight patients (20.5%), and 2 in three patients (7.7%).

### Treatment

The median number of treatment cycles was 8.0 (interquartile range, 6.0–14.5; range, 1.0–67.0). IRI treatment interruption occurred in 10 patients (25.6%), discontinuation in one patient (2.6%), and dose reduction in 10 patients (25.6%). For RAM, treatment interruption occurred in 10 patients (25.6%), discontinuation in five patients (12.8%), and dose reduction in three patients (7.7%). The mean RDI was 87.8% ± 20.11 for IRI and 87.6% ± 25.33 for RAM, indicating that a relatively high RDI was maintained for both agents (Online Resource 3).

### Tumor response

Among the 26 patients with measurable lesions, two achieved a CR, eight achieved a PR, and 10 had stable disease. Accordingly, the ORR was 38.5% (95% CI: 20.2–59.4%), and the DCR was 76.9% (95% CI: 56.4–91.0%) (Online Resource 2). The maximum percentage changes in tumor size from baseline are shown in Fig. 1.

### Survival

**Fig. 2:**
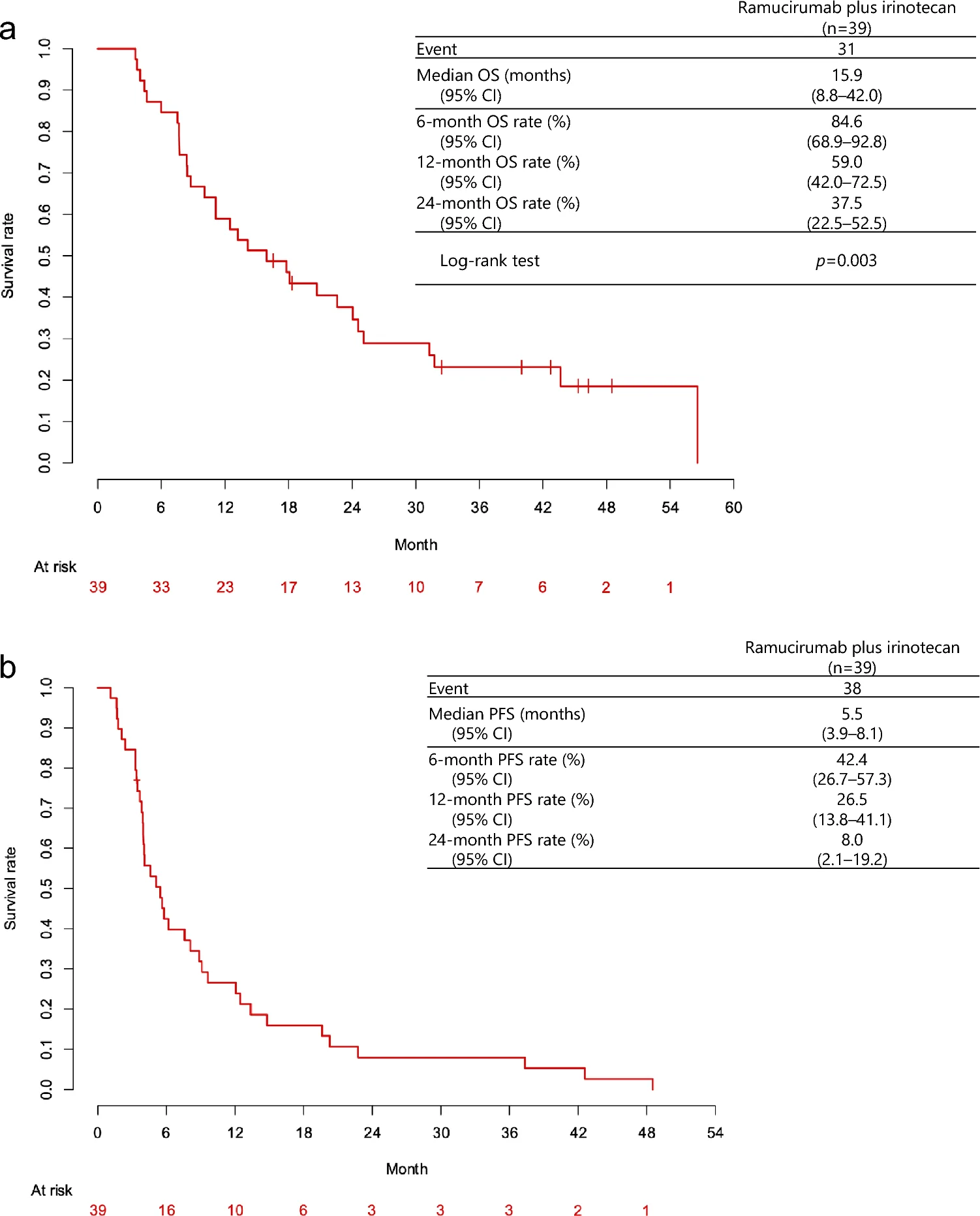
Kaplan–Meier curves Kaplan-–Meier curves of (A) overall survival (OS) and (B) progression-free survival (PFS) in patients with advanced gastric cancer receiving ramucirumab plus irinotecan. Abbreviations: CI, confidence interval

The median follow-up period was 15.9 months (mean: 19.9; maximum: 56.6). The median OS was 15.9 months (95% CI: 8.2–42.0; 90% CI: 11.1–24.6). The 6-month, 1-year, and 2-year OS rates were 84.6% (95% CI: 68.9–92.8), 59.0% (95% CI: 42.0–72.5), and 37.5% (95% CI: 22.5–52.5), respectively (Fig. 2a). In a one-sample log-rank test with a threshold median OS of 7 months, the observed OS was significantly longer than the threshold (*p* = 0.003). The median PFS was 5.5 months (95% CI: 3.9–8.1). The 6-month, 1-year, and 2-year PFS rates were 42.4% (95% CI: 26.7–57.3), 26.5% (95% CI: 13.8–41.1), and 8.0% (95% CI: 2.1–52.19.2), respectively (Fig. 2b). The median TTF was 4.1 months (95% CI: 3.7–5.8). The 3-month, 6-month, and 1-year TTF rates were 76.9% (95% CI: 60.3–87.3), 33.3% (95% CI: 19.3–48.0), and 15.4% (95% CI: 6.2–28.3), respectively (Online Resource 4).

### Subsequent treatments

Among the 39 eligible patients who received protocol treatment, 33 patients (84.6%) received post-study treatment, whereas 6 patients (15.4%) did not. Of the 33 patients who received subsequent treatment, 32 (97.0%) underwent further chemotherapy, 4 (12.1%) received radiotherapy, and 2 (6.1%) underwent surgery. Among the 32 patients who received post-study chemotherapy, 13 (40.6%) received one regimen, 13 (40.6%) received two regimens, 4 (12.5%) received three regimens, and 2 (6.2%) received four regimens. The most frequently administered subsequent chemotherapy was nivolumab (n = 21, 63.6%), followed by FTD/TPI (n = 7, 21.2%), CAPOX plus nivolumab (n = 5, 15.6%), and paclitaxel plus ramucirumab (n = 4, 12.1%). Other regimens included irinotecan (n = 3, 9.1%), XELOX (n = 3, 9.1%), SOX (n = 2, 6.1%), nab-paclitaxel (n = 2, 6.1%), irinotecan plus ramucirumab (n = 2, 6.1%), TAS-102 (n = 1, 3.0%), futibatinib (n = 1, 3.0%), CPT-11 plus ramucirumab (n = 1, 3.0%), weekly paclitaxel plus ramucirumab (n = 1, 3.0%), SOX plus nivolumab (n = 1, 3.0%), paclitaxel (n = 1, 3.0%), FTD/TPI plus ramucirumab (n = 1, 3.0%), FOLFOX plus nivolumab (n = 1, 3.0%), and investigational treatment (n = 1, 3.0%).

### Subgroup analysis for OS

In subgroup analyses, patients with an mGPS of 1–2 had significantly shorter OS than those with an mGPS of 0 (median OS: 14.2 vs. 18.1 months; HR: 2.545; 95% CI: 1.156–5.603; *p* = 0.020), indicating that a higher mGPS was a poor prognostic factor. Patients with a PNI ≥ 44.91 tended to have longer OS than those with a PNI < 44.91 (median OS: 24.1 vs. 13.2 months; HR: 0.621; 95% CI: 0.302–1.281; *p* = 0.197). Tumor shrinkage according to RECIST was strongly associated with favorable outcomes. Patients achieving CR or PR had significantly longer OS than those with stable or progressive disease (median OS: 31.3 vs. 8.4 months; HR: 0.350; 95% CI: 0.134–0.913; *p* = 0.031). Similarly, when patients were stratified into disease control (CR/PR/stable disease) versus progressive disease, OS was markedly longer in the disease control group (median OS: 24.1 vs. 6.0 months; HR: 0.109; 95% CI: 0.035–0.341; *p* < 0.001), indicating that both tumor response and disease control were significant predictors of improved survival (Table 2).

**Table 2:** Prognostic factors for OS

		n	Median OS	HR [95% CIs]	*p*
ECOG PS	0	24	16.1 [8.4, 31.8]	0.699 [0.338, 1.445]	0.333
	1	15	15.9 [7.5, 22.6]		
Recurrence site	= 1	26	18.1 [12.5, 31.3]	0.660 [0.311, 1.404]	0.281
	≥ 2	13	8.4 [7.5, 22.6]		
UGT1A1	Wild	15	22.6 [7.7, 31.8]	0.686 [0.295, 1.595]	0.381
	Single hetero	13	11.1 [4.6, 25.1]		
Pathological subtype	Intestinal	15	15.9 [8.8, NA]	0.695 [0.325, 1.488]	0.348
	Diffuse	24	14.5 [7.7, 25.1]		
Time of recurrence	After adjuvant DS	12	15.7 [8.4, NA]	0.652 [0.289, 1.472]	0.303
	During adjuvant DS	27	15.9 [7.7, 24.6]		
NLR (median)	≥ 1.875	20	12.9 [7.5, 31.3]	1.173 [0.571, 2.411]	0.664
	< 1.875	19	20.7 [8.4, 25.1]		
PNI (median)	≥ 44.91	20	24.1 [8.4, 43.7]	0.621 [0.302, 1.281]	0.197
	< 44.91	19	13.2 [7.5, 20.7]		
mGPS	1~2	11	14.2 [3.7, 20.7]	2.545 [1.156, 5.603]	0.020
	0	28	18.1 [10.1, 43.7]		
RECIST (1)	CR/PR	11	31.3 [13.2, NA]	0.350 [0.134, 0.913]	0.031
	SD/PD	15	8.4 [4.4, 15.9]		
RECIST (2)	CR/PR/SD	19	24.1 [11.1, NA]	0.109 [0.035, 0.341]	<0.001
	PD	7	6.0 [3.5, 8.4]		

CR, complete response; PR, partial response; SD, stable disease; PD, progressive disease; CI, confidence interval; ECOG PS, Eastern Cooperative Oncology Group Performance Status; HR, hazard ratio; mGPS, modified Glasgow Prognostic Score; NLR, neutrophil-to-lymphocyte ratio; OS, overall survival; PNI, Prognostic Nutritional Index; RECIST, Response Evaluation Criteria in Solid Tumors; UGT1A1, UDP-glucuronosyltransferase family 1 member A1; NA, Not Available

### Safety

The incidences of treatment-related adverse events are summarized in Table 3. Grade 3/4 hematological adverse events included neutropenia in 10 patients (25.6%), leukopenia in four (10.3%), thrombocytopenia in one (2.6%), hypoalbuminemia in one (2.6%), and increased AST and ALT levels in two patients each (5.1%). Grade 3/4 non-hematological adverse events included hypertension in 10 patients (25.6%), anorexia in three (7.7%), febrile neutropenia in three (7.7%), nausea in one (2.6%), vomiting in one (2.6%), and malaise in one patient (2.6%). No treatment-related deaths or new safety signals were observed.

**Table 3:** Adverse events

Adverse event	Ramucirumab plus irinotecan (n = 39)	
	Any grade	Grades 3–4
Leukopenia	18 (46.2%)	4 (10.3%)
Neutropenia	27 (69.2%)	10 (25.6%)
Thrombocytopenia	14 (35.9%)	1 (2.6%)
Anemia	35 (89.7%)	0 (0.0%)
Hypoalbuminemia	34 (87.2%)	1 (2.6%)
Proteinuria	22 (56.4%)	0 (0.0)
AST increased	24 (61.5%)	2 (5.1%)
ALT increased	21 (53.8%)	2 (5.1%)
Creatinine increased	5 (12.8%)	1 (2.6%)
Diarrhea	27 (69.2%)	2 (5.1%)
Oral mucositis	5 (12.8%)	0 (0.0%)
Nausea	13 (33.3%)	1 (2.6%)
Vomiting	5 (12.8%)	1 (2.6%)
Malaise	22 (56.4%)	1 (2.6%)
Anorexia	22 (56.4%)	3 (7.7%)
Alopecia	12 (30.8%)	0 (0.0%)
Febrile neutropenia	3 (7.7%)	3 (7.7%)
Hypertension	25 (64.1%)	10 (25.6%)

ALT, alanine aminotransferase; AST, aspartate aminotransferase

## Discussion

This phase II RAMIEL trial demonstrated that RAM–IRI showed clinically meaningful efficacy and manageable safety in patients with GC who experienced early recurrence during or within 6 months after completion of adjuvant DS chemotherapy. The median OS of 15.9 months was not only significantly longer than the predefined threshold of 7 months and met the primary endpoint, but was also notably longer than the expected value of 11 months. These findings suggest that the RAM–IRI regimen is a promising treatment option for this population with limited second-line treatment options.

The Gastric Cancer Treatment Guidelines 2018, published by the Japanese Gastric Cancer Association, do not recommend the re-administration of agents already used in the adjuvant setting for patients who experience relapse during or within 6 months after adjuvant chemotherapy [12]. This recommendation is supported by a retrospective analysis by Shitara et al., which demonstrated that S-1 plus cisplatin was largely ineffective in patients with early recurrence, particularly those with a relapse-free interval (RFI) of < 6 months after adjuvant S-1 [13]. Outcomes were markedly poorer in patients with an RFI of < 6 months than in those with later recurrence (median PFS: 2.3 vs. 6.2 months; median OS: 7.3 vs. 16.6 months). Early recurrence within 6 months after DS therapy is consistently associated with resistance to both fluoropyrimidines and taxanes, leaving few effective therapeutic options. The clinical relevance of maintaining S-1 beyond progression was assessed in the JACCRO-GC05 and CCOG0701 trials, in which IRI and paclitaxel, respectively, were administered in combination with S-1 following failure of first-line S-1 plus cisplatin [20, 21]. Both trials consistently showed no advantage of continued S-1 administration beyond progression—a conclusion that can be extrapolated to patients who relapse during or within 6 months after completion of adjuvant S-1–based chemotherapy. Similarly, a phase II trial of capecitabine plus cisplatin for GC showed consistently poorer outcomes in patients with an RFI of ≤ 6 months than in those with an RFI of > 6 months, including lower response rates (21% vs. 39%, *p* = 0.427), shorter TTF (5.4 vs. 8.3 months, *p* = 0.072), and reduced OS (9.3 vs. 14.1 months, *p* = 0.075) [22]. Comparatively, the survival outcomes in our trial appear favorable and provide the first prospective evidence that the RAM–IRI regimen can overcome this therapeutic deadlock, particularly in East Asian countries where adjuvant DS chemotherapy is widely used for stage III GC. In the current biomarker-driven treatment era, platinum-based regimens combined with immunotherapy or targeted agents remain important first-line options for advanced GC, especially for patients with actionable biomarkers such as HER2, PD-L1, MSI-H/dMMR, or CLDN18.2 [23–25]. However, their role in patients who experience recurrence during or within 6 months after S-1-based adjuvant therapy remains unclear, because such early relapse likely reflects aggressive tumor biology and reduced sensitivity to prior fluoropyrimidine-based treatment. Therefore, although biomarker-matched platinum-based regimens may still be appropriate for selected patients, RAM–IRI may be a reasonable alternative for patients without actionable biomarkers or for those with fluoropyrimidine-refractory early relapse, as it avoids re-use of the adjuvant backbone and provides a taxane-free treatment option. In addition, a phase II trial of CapeOX plus nivolumab for HER2-negative gastric cancer with early relapse during or after adjuvant S-1 or DS therapy (JACCRO GC-11: FirSTAR trial) is currently ongoing, and its results are awaited to clarify the potential role of immune checkpoint inhibitor plus oxaliplatin-based therapy in this setting [26].

Neoadjuvant or perioperative strategies are widely employed in Western countries, particularly in Europe, where the docetaxel, oxaliplatin, leucovorin, and 5-fluorouracil regimen combined with durvalumab (FLOT plus durvalumab) has demonstrated significant survival benefits compared with the FLOT regimen alone in patients with locally advanced GC [27–30]. However, clinical data remain limited for patients with early GC recurrence even after perioperative FLOT plus durvalumab, underscoring the need for effective subsequent chemotherapy regimens other than paclitaxel because of the potential for cross-resistance between docetaxel and paclitaxel. The phase II RAMIRIS trial investigated the efficacy of RAM plus FOLFIRI compared with paclitaxel plus RAM as second-line therapy in advanced or metastatic gastroesophageal adenocarcinoma, including patients who relapsed after FLOT [17]. In that trial, the RAM plus FOLFIRI arm demonstrated superior activity compared with paclitaxel plus RAM, with an ORR of 25% versus 8% and a median PFS of 4.6 versus 2.1 months. Collectively, these data suggest that RAM plus FOLFIRI may be a promising taxane-free second-line strategy for patients with GC and prior exposure to docetaxel. The RAM plus IRI regimen, which does not exhibit cross-resistance to key agents used in perioperative chemotherapy, may therefore represent a promising treatment option for early recurrence following perioperative FLOT plus durvalumab. As peripheral venous access ports are generally unnecessary, the port-free RAM–IRI regimen represents a more convenient alternative to FOLFIRI. Furthermore, the clinical relevance of our findings may extend beyond the Japanese treatment paradigm. Early recurrence after perioperative FLOT-based therapy, particularly when combined with immune checkpoint inhibitors, also likely reflects aggressive tumor biology and resistance to prior cytotoxic agents. In this context, treatment strategies that avoid cross-resistance to taxanes are of particular importance. Given the activity of irinotecan-based, anti-angiogenic regimens in taxane-pretreated populations, as suggested by the RAMIRIS trial, RAM–IRI may also represent a rational treatment option for patients who experience early relapse after perioperative FLOT-based therapy.

In the present study, mGPS was identified as a significant prognostic factor for overall survival. The mGPS has been recognized as a useful marker reflecting cancer cachexia and tumor burden, whereas the PNI is generally considered an indicator of perioperative nutritional status [31–34]. Because all patients in this study had undergone gastrectomy and subsequently developed early recurrence, overall survival in this population may have been influenced more strongly by the impact of progressive recurrent disease, including cancer-related systemic inflammation and cachexia, than by postoperative nutritional status alone. This may partly explain why mGPS, but not PNI, was significantly associated with overall survival in the present analysis. In clinical practice, these markers may still be useful for risk assessment and supportive care planning; however, given the limited sample size, further studies are needed to clarify their clinical relevance.

Regarding safety, the toxicity profile of RAM plus IRI in our study was consistent with previous reports, with no unexpected safety signals. The most frequent grade ≥ 3 hematological adverse event was neutropenia (25.6%), followed by leukopenia (10.3%) and febrile neutropenia (7.7%). Among non-hematological events, hypertension (25.6%), anorexia (7.7%), and diarrhea (5.1%) were the most common and were generally manageable with supportive care and dose modification; no treatment-related deaths occurred. The RDI was maintained at 87.8% for IRI and 87.6% for RAM, suggesting that the regimen was well tolerated overall. Thus, RAM–IRI provides a clinically feasible treatment option with an acceptable safety profile for patients with early recurrence after adjuvant DS therapy.

This study has some limitations. First, it was a single-arm phase II trial with a small sample size, which may limit generalizability. Second, the trial exclusively included patients with early recurrence after adjuvant DS therapy, which may not fully reflect treatment patterns in regions where perioperative regimens such as FLOT are commonly used. Third, the absence of a randomized control arm precluded direct comparison of RAM–IRI with other available regimens. Finally, key treatment-related biomarkers, including CLDN18.2, PD-L1, and MSI, were not evaluated, and neither exploratory biomarker analyses nor quality-of-life assessments were performed.

In conclusion, RAM–IRI demonstrated promising efficacy and manageable toxicity in patients with early GC recurrence following adjuvant DS chemotherapy. This combination regimen therefore constitutes a favorable treatment option for patients with GC refractory to both fluoropyrimidine- and docetaxel-based therapies.

## Supplementary Information

Below is the link to the electronic supplementary material.

Supplementary file1 (DOCX 37 KB)

Supplementary file2 (DOCX 19 KB)

Supplementary file3 (DOCX 19 KB)

Supplementary file4 (DOCX 41 KB)

## Data Availability

The datasets generated and/or analyzed during the current study are not publicly available because of ethical restrictions and the requirement to protect patient confidentiality. In accordance with the approved study protocol and institutional review board regulations, individual patient-level data cannot be shared.
