# Supplementary material for: A multicenter phase II trial of ramucirumab plus irinotecan for early recurrence of gastric cancer during or after adjuvant chemotherapy with docetaxel plus S-1 therapy: the RAMIEL trial (OGSG1901)
Source: Gastric Cancer. 2026 Sep 16;29(6):1349–59. doi: 10.1007/s10120-026-01799-8 (PMC13754997; doi:10.1007/s10120-026-01799-8)
Supplement: Supplementary file 2 [file 10120_2026_1799_MOESM2_ESM.docx]

*Gastric Cancer*

**A Multicenter Phase II Trial of Ramucirumab Plus Irinotecan for Early Recurrence of Gastric Cancer During or After Adjuvant Chemotherapy with Docetaxel Plus S-1 Therapy: The RAMIEL Trial (OGSG1901)**

Toshifumi Yamaguchi^a^, Ryohei Kawabata^b^, Yusuke Akamaru^c^, Hidekazu Hirano^d^, Keijiro Sugimura^e^, Yasuhiro Miyazaki^f^, Yoshihiro Kakeji^g^, Yosuke Kito^h^, Takashi Nomura^i^, Takeshi Kawakami^j^, Masahiro Tanemura^k^, Atsushi Takeno^l^, Hiroyuki Okuda^m^, Tomohiro Matsushima^n^, Toshio Shimokawa^o^, Yukinori Kurokawa^p^, Shunji Endo^q^, Narikazu Boku^r^, Taroh Satoh^s^

^a^ Cancer Chemotherapy Center, Osaka Medical and Pharmaceutical University Hospital,

2-7, Daigaku-machi, Takatsuki, Osaka,569-8686, Japan; yamagu.toshifumi@gmail.com

^b^ Department of Surgery, Sakai City Medical Center, 1-1-1 Ebaraji-cho, Sakai-shi, Osaka, 272-1199, Japan; helloryoheikawabata@gmail.com

^c^ Department of Gastroenterological Surgery, Osaka Rosai Hospital, 1179-3 Nakasonecho, Kita-ku, Sakai-shi, Osaka 591-8025, Japan; y-akamaru@osakah.johas.go.jp

^d^ Department of Gastrointestinal Medical Oncology, National Cancer Center Hospital, 5-1-1 Tsukiji, Chuo-ku, Tokyo, 104-0045, Japan; hihirano@ncc.go.jp

^e^ Department of Surgery, Kansai Rosai Hospital, 3-1-69 Inabaso, Amagasaki, Hyogo 660-8511, Japan; sugimura-keijiro@kansaih.johas.go.jp

^f^ Department of Surgery, Osaka General Medical Center, 3-1-56 Bandaihigashi, Sumiyoshi-ku, Osaka city, OSAKA 558-8558, Japan; miyazakiya@gh2.opho.jp

^g^ Division of Gastrointestinal Surgery, Department of Surgery, Kobe University Graduate School of Medicine, 7-5-1 Kusunoki-cho, Chuo-ku, Kobe 650-0017, Japan; kakeji@med.kobe-u.ac.jp

^h^ Department of Medical Oncology, Ishikawa Prefectural Central Hospital, 2-1 Kuratsuki Higashi, Kanazawa City, Ishikawa Prefecture 920-8530, Japan; kitoyo9100@gmail.com

^i^ Department of Surgery, Yamagata Prefectural Central Hospital, 1800 Aoyanagi, Yamagata-shi, Yamagata 990-2292, Japan; tnomura@ypch.gr.jp

^j^ Division of Gastrointestinal Oncology, Shizuoka Cancer Center, 1007 Shimonagakubo, Nagaizumi-cho, Sunto-gun, Shizuoka 411-8777, Japan; t.kawakami@scchr.jp

^k^ Department of Surgery, Rinku General Medical Center, 2-23 Rinku Orai-Kita, Izumisano-City, Osaka, 598-8577, Japan; m-tanemura@rgmc.izumisano.osaka.jp

^l^ Department of Surgery, NHO Osaka National Hospital, 2-1-14 Hoenzaka, Chuo-ku, Osaka, Osaka, 540-0006 Japan; takeno.atsushi.pk@mail.hosp.go.jp

^m^ Department of Medical Oncology, Keiyukai Sapporo Hospital, 1-1, Minami 9-chome, Hondori, Shiroishi-ku, Sapporo-shi, Hokkaido 003-0026, Japan; hiokuda@keiyukaisapporo.or.jp

^n^ Department of Gastroenterology, Saitama Cancer Center, 818 Komuro, Ina-machi, Kitaadati-gun, Saitama 362-0806, Japan; t.matsushima@saitama-pho.jp

^o^ Department of Biostatistics, Faculty of Medicine, Wakayama Medical University, 811-1 Kimiidera, Wakayama-shi, 641-8509, Japan; toshibow2000@gmail.com

^p^ Department of Gastroenterological Surgery, Graduate School of Medicine, The University of Osaka, 2-2 Yamadaoka, Suita 565-0871, Japan; ykurokawa@gesurg.med.osaka-u.ac.jp

^q^ Department of Digestive Surgery, Kawasaki Medical School, 577 Matsushima, Kurashiki City, Okayama 701-0192, Japan; endo-s@med.kawasaki-m.ac.jp

^r^ Department of Oncology and General Medicine, IMSUT Hospital, Institute of Medical Science, University of Tokyo, 4-6-1, Shirokanedai, Minato-ku, Tokyo 108-8639, Japan; nboku@ims.u-tokyo.ac.jp

^s^ Center for Cancer Genomics and Precision Medicine, The University of Osaka Hospital, 2-2 Yamadaoka, Suita 565-0871, Japan; taroh@cfs.med.osaka-u.ac.jp

***Corresponding author:** Narikazu Boku

IMSUT Hospital, Institute of Medical Science, University of Tokyo,

4-6-1, Shirokanedai, Minato-ku, Tokyo 108-8639, Japan

Tel: +81-3-5449-5320

Email: nboku@ims.u-tokyo.ac.jp

Online Resource 2. Summary of Sites of recurrence

| **Sites of recurrence** | **n (%)** |
| --- | --- |
| Lymph nodes | 16 (41.0) |
| Peritoneum | 8 (20.5) |
| Liver | 2 (5.1) |
| Lung | 1 (2.6) |
| Lymph nodes + peritoneum | 4 (10.3) |
| Lymph nodes + liver | 1 (2.6) |
| Lymph nodes + liver + bone | 1 (2.6) |
| Lymph nodes + bone | 1 (2.6) |
| Peritoneum + ovary | 1 (2.6) |
| Peritoneum + lung | 2 (5.1) |
| Liver + bone | 1 (2.6) |
| Lung + pleura + carcinomatous lymphangitis | 1 (2.6) |
